# Heart Disease in Children

**Published:** 1890-09-06

**Authors:** 


### HEART DISEASE IN CHILDREN.

Dr. Mitchell Bruce's address before the Cardiff Medical Society on disease of the heart in children has been recently published. The anatomy and physiology of the circulation are peculiar in young subjects, and the causes of cardiac disease differ from those of heart affections in older subjects. Then there is physical and mental strain, and various cardiac poisons. The symptons are also modified by age. Acute rheumatism is in Dr. Bruce's opinion the most common cause of acute inflammation of the heart in the child. But rheumatism is frequently latent in the child. We would ask why, therefore, it should be called acute? Dr. Bruce says in every case of pyrexia in young subjects with obscure pains in the limbs, "growing pains" as they are called, the stethoscope should be used frequently. Four tests may be employed. Stethoscopic examination; effect of anti-rheumatic treatment, especially of salicylates; discovery after minute examination of tenderness of joints; investigation of family history. The peculiarity of the symptoms is relative latency or mildness, as compared with symptoms in adults; the second clinical feature is the great susceptibility of children to impressions. Slight causes raise the temperature and aggravate the malady. As regards complications, pulmonary disease is more common in children than in adults. With reference to examination, Dr. Bruce reminds us that the heart lies higher in the thorax of the child, the apex-beat being in the fourth space, and more to the left than in the adult. The question of the ultimate prognosis of acute heart disease in the child is also considered. Dr. Bruce considers it is better in the child than in the adult, that the probability of permanent valve disease is less, and that in a number of cases the signs of valvular disease ultimately disappear. But the child may be left with chronic valvular disease as mitral regurgitation, or with adherent pericardium, or with both. Then compensation has to be established. This must be effected by careful and good dieting, by avoiding either physical or mental strain, and by special protection from rheumatism.

